# Polyphenol-Mediated Gut Microbiota Modulation: Toward Prebiotics and Further

**DOI:** 10.3389/fnut.2021.689456

**Published:** 2021-06-28

**Authors:** Maria Carolina Rodríguez-Daza, Elena C. Pulido-Mateos, Joseph Lupien-Meilleur, Denis Guyonnet, Yves Desjardins, Denis Roy

**Affiliations:** ^1^Faculty of Agriculture and Food Sciences, Institute of Nutrition and Functional Foods (INAF), Laval University, Québec, QC, Canada; ^2^Department of Food Science, Faculty of Agriculture and Food Sciences, Laval University, Québec, QC, Canada; ^3^Diana Nova, Symrise Nutrition, Clichy-la-Garenne, France; ^4^Department of Plant Science, Faculty of Agriculture and Food Sciences, Laval University, Québec, QC, Canada

**Keywords:** polyphenols, prebiotics, antimicrobial action, polyphenol-associated enzymes, *Akkermansia muciniphila*, *duplibiotic*, *Lactiplantibacillus plantarum*, trophic interactions

## Abstract

The genome of gut microbes encodes a collection of enzymes whose metabolic functions contribute to the bioavailability and bioactivity of unabsorbed (poly)phenols. Datasets from high throughput sequencing, metabolome measurements, and other omics have expanded the understanding of the different modes of actions by which (poly)phenols modulate the microbiome conferring health benefits to the host. Progress have been made to identify direct prebiotic effects of (poly)phenols; albeit up to date, these compounds are not recognized as prebiotics *sensu stricto*. Interestingly, certain probiotics strains have an enzymatic repertoire, such as tannase, α-L-rhamnosidase, and phenolic acid reductase, involved in the transformation of different (poly)phenols into bioactive phenolic metabolites. *In vivo* studies have demonstrated that these (poly)phenol-transforming bacteria thrive when provided with phenolic substrates. However, other taxonomically distinct gut symbionts of which a phenolic-metabolizing activity has not been demonstrated are still significantly promoted by (poly)phenols. This is the case of *Akkermansia muciniphila*, a so-called antiobesity bacterium, which responds positively to (poly)phenols and may be partially responsible for the health benefits formerly attributed to these molecules. We surmise that (poly)phenols broad antimicrobial action free ecological niches occupied by competing bacteria, thereby allowing the bloom of beneficial gut bacteria. This review explores the capacity of (poly)phenols to promote beneficial gut bacteria through their direct and collaborative bacterial utilization and their inhibitory action on potential pathogenic species. We propose the term *duplibiotic*, to describe an unabsorbed substrate modulating the gut microbiota by both antimicrobial and prebiotic modes of action. (Poly)phenol *duplibiotic* effect could participate in blunting metabolic disturbance and gut dysbiosis, positioning these compounds as dietary strategies with therapeutic potential.

## Introduction

The gut microbiota plays a crucial role on host physiology. It is well-known that an intimate symbiotic relationship exists between the gut microbiome and the host, and that this association is complex and multi-dimensional, as it affects the gut-lung, gut-brain, gut-skin, gut-muscle, and gut-adipose tissue axes, among others (1–4). The tight connection between the gut ecosystem and host endogenous metabolic responses is receiving much attention lately, owing to its key involvement in the onset and progress of cardiometabolic diseases (4), intestinal inflammation (5), cancer (6), cognition (7), and neuropsychological disorders (1). The gut microbiota is now considered a relevant therapeutic target for many chronic societal diseases.

Diet plays a dominant role in modulating the intestinal microbiota (8). The fermentation of its components by commensal microbiota generates an array of cross-feeding networks which provide the host with nutrients and chemical signals affecting both immunity and metabolism (9–11). Many of these dietary substrates are prebiotics, which are selectively utilized by host colonic microbes conferring health benefits (12) by promoting the growth and the activity of beneficial bacterial strains. Until recently, the prebiotic concept was confined to selected non-digestible carbohydrates; however, phytochemicals, such as (poly)phenols, exert potentially prebiotic effects by selectively stimulating beneficial bacteria and reducing the incidence of diseases (12). (Poly)phenols are a diverse class of secondary plant metabolites found in most diets. Owing to their chemical structure, they are poorly absorbed and thus reach the colon, where they affect the resident microbiota (13, 14). Specifically, (poly)phenols can stimulate several of keystone bacterial species such as *Akkermansia muciniphila, Bacteroides thetaiotaomicrom, Faecalibacterium prausnitzii*, Bifidobacteria, and Lactobacilli (15–20).

The beneficial direct impact of (poly)phenols on the gut microbiota relies on two principal modes of action: a direct bacterial stimulatory effect and a direct antimicrobial effect. As a corollary to carbohydrate-associated enzymes (CAZymes) used by prebiotic stimulated bacteria, the genome of several beneficial bacteria encodes an array of (poly)phenol-associated enzymes (PAZymes) involved specifically in (poly)phenol metabolization (21, 22). In the presence of (poly)phenols, PAZymes-producing bacteria can utilize these compounds to improve their fitness and their persistence in intestinal niches. In addition, (poly)phenols can also selectively inhibit the development of potential pathogenic species often associated with metabolic disorders (5, 23–26).

The direct effects of (poly)phenols on the microbiota engender bacterial ecological shifts and favor syntrophic relationships that further modulate microbiota's composition and function. Indeed, collaterally to (poly)phenols antimicrobial effect, (poly)phenol resistant bacteria, such as *Akkermansia muciniphila*, are boosted after dietary (poly)phenol intake, highlighting their capacity to withstand the (poly)phenol's antimicrobial action and their ability to opportunistically occupy freed ecological niches. Furthermore, through successive catabolic reactions, PAZymes producing bacteria can provide phenolic metabolites used by other beneficial bacterial species in complex trophic cross-feeding chains. In this sense, emerging reports suggest that certain (poly)phenols can be utilized and transformed by beneficial gut microbes, such as *Lactiplantibacillus plantarum* (10, 27, 28), into bioactive phenolic metabolites that are later freely absorbed and transported to target organs. In this respect, it appears that some (poly)phenols may exert a genuine prebiotic effect by stimulating commensal bacteria and by inducing the production of (poly)phenol beneficial metabolites, further contributing to human health.

This review discusses how (poly)phenols interact directly with the gut microbiota to provide benefits to the host. To account for the two main (poly)phenols modes of action, we suggest the use of the term “*duplibiotics*” which broadens the scope of prebiotic activity to include and define a substrate able to modulate the gut microbiota's composition through a dual antimicrobial effect and beneficial bacteria stimulatory effect. To explore this concept, we consider the inhibitory activity of (poly)phenols on diet-induced inflammatory bacteria often associated with obesity and discuss their stimulatory action on several commensal bacterial species. We also provide insights on key bacterial enzymes involved in the potential prebiotic effects of (poly)phenols. These enzymes release (poly)phenol metabolites which participate in intricate microbial trophic networks in the intestinal environment. Finally, relying on *A. muciniphila* as a model organism, we describe how (poly)phenol-induced ecological changes can promote the growth of symbionts that are less metabolically adapted for the transformation of (poly)phenols.

## The Gut Microbiota and Its Involvement in Host Health

The human gastrointestinal (GI) tract is inhabited by about 10^14^ metabolically active bacteria (29). The gut microbiota is mainly represented by a limited number of bacterial phyla: *Firmicutes, Bacteroidota* (30) (accounting for ~90% of the GI microbiota), *Actinobacteria, Proteobacteria*, and *Verrucomicrobia* (21, 22). Despite high inter-individual variability, *Firmicutes* is the largest phylum in humans and rodents, with more than 250 genera (22, 31). Moreover, the phylum *Bacteroidota* regroups around 20 genera, among which the genus *Bacteroides* is the most represented (31). The phylum *Actinobacteria* is less consistently detected as dominant and represents a lower percentage (5%) of the total bacteria; it includes the genus *Bifidobacterium* from which many probiotics have been identified (32). Finally, the phylum *Verrucomicrobia* includes the genus *Akkermansia* spp., which is present in low proportion (3–5%) and is now considered a next-generation probiotic (32, 33). On average, the gut microbiota encodes about 40 times more genes than its human host (34), providing an extended metabolic capacity largely oriented toward the catabolism of complex diet-acquired compounds.

The gut microbiota is particularly specialized in the degradation of plant constituents with thousands of genes dedicated to the digestion of complex carbohydrates (35). Apart from glycans, the gut microbiota also ferments and synthesizes proteins, transforms xenobiotics, as well as host bile acids, and provides the body with essential vitamins (36, 37). This intense activity leads to the production of beneficial bacterial metabolites such as short-chain fatty acids (SCFA, mainly acetate, propionate, and butyrate), indoles, neurotransmitters, and gasotransmitters (ex. H_2_S, NO) (38). The SCFA are tightly involved in energy homeostasis, insulin response, fat accumulation, and immune signaling, as reviewed elsewhere (39, 40). Among the SCFA, butyrate reinforces the intestinal mucosal barrier (41, 42), suppresses proinflammatory effectors in macrophages, and promotes the differentiation of dendritic cells (9). Moreover, gut microbial activity can also produce harmful metabolites such as several secondary bile acids, p-cresol, p-tyramine, trimethylamine-N-oxide [recently reviewed by McCarville et al. (38), Cortes-Martin et al. (43), and Saito et al. (44)].

The colonic epithelium is associated to an extensive lymphoid tissue representing the majority of the body's immunocytes (45) which constantly senses the commensal gut microbiota. The immune receptors on intestinal epithelial or on dendritic cells survey the microbial structures known as “pathogen recognition patterns,” and can signal the presence of pathogens to initiate an immune response (46). Under normal physiological conditions, the gut microbiota is maintained in a healthy homeostatic state (i.e., eubiosis) and preserves a balanced local gut immunological interaction with the host. An imbalance in the composition, diversity and metabolic capacity of the gut microbiota (dysbiosis) (47) can, however, negatively affect the genes (meta-transcriptomes), proteins (metaproteomes), functions (metagenomes), and metabolites (metabolomes) makeup, thereby influencing the whole intestinal environment (microbiome) and host health. Gnotobiotic murine models, fecal transplant experiments, or human twins' studies, have clearly demonstrated that an altered crosstalk between the gut microbiota and the host mucosal immune system is involved in the etiology of several chronic metabolic diseases (5, 25, 48, 49).

Numerous studies have highlighted the capacity of specific bacterial phylotypes in triggering beneficial immunological responses and reducing the severity of inflammatory diseases. This is the case of Bifidobacteria and Lactobacilli species, which attenuate intestinal inflammation and metabolic disturbances in ulcerative colitis (50, 51) and obesity (52, 53). For instance, certain probiotic strains, such as *L. plantarum* WCFS1, improve inflammation by lowering the levels of plasma proinflammatory cytokines (51). Other relevant microorganisms considered as next-generation probiotics like *A. muciniphila, B. thetaiotaomicron*, and *F. prausnitzii* are also intricately interacting with the host immune system and have been associated with beneficial effects on the host (54–56).

**Figure 1:**
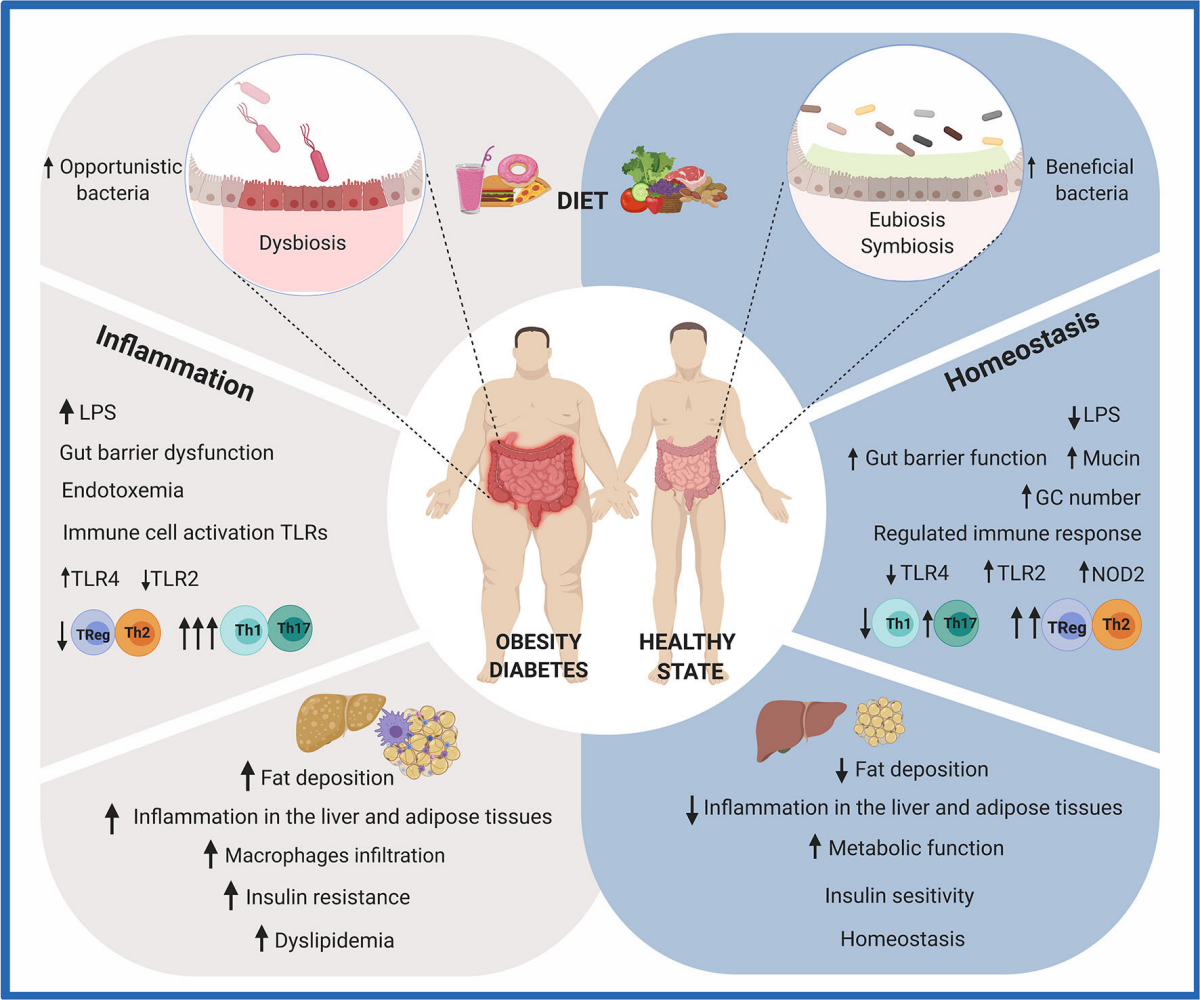
Effects of diet on gut health. The consumption of a diet rich in fat and sugar (HFHS) has been shown to negatively modulate the composition and metabolic activity of the human gut microbiota. Due to the crucial role it plays in human health, imbalances in gut microbiota composition and/or function (dysbiosis) are recognized as possible causes of intestinal, metabolic, and immune diseases. Particularly, HFHS leads to the translocation of bacterial lipopolysaccharides (LPS) and chronic inflammation. A group of metabolic abnormalities, including fat deposition, insulin resistance, hyperglycemia, and dyslipidemia, is exacerbated. On the other hand, a diet rich in fruits and vegetables contributes to gut homeostasis. Dietary functional ingredients have been shown to increase microbial diversity and functions, maintaining the gut microbiota composition in a eubiotic state. Intestinal and immune homeostasis is positively modulated in the host. This figure was created with BioRender.com.

All in all, an increasing number of studies demonstrate the biological involvement of the gut microbiota in the onset, maintenance, or progress of different chronic diseases (6, 57–61). It is therefore of particular interest to identify the composition and the functional contribution of the gut microbiota to assess mechanisms by which they improve the host health. This knowledge is of paramount importance to develop microbiota-based therapies, including non-pharmaceutical interventions, such as dietary functional ingredients (prebiotics), live microbes (probiotics and fermented food), or microbial metabolites. The effect of diet on the gut microbiota and its influences on the host immunological responses and metabolic phenotype are summarized in Figure 1.

## Reshaping the Gut Microbiota With (Poly)phenol-Enriched Diets: Introducing the Concept of *Duplibiotics*

**Figure 2:**
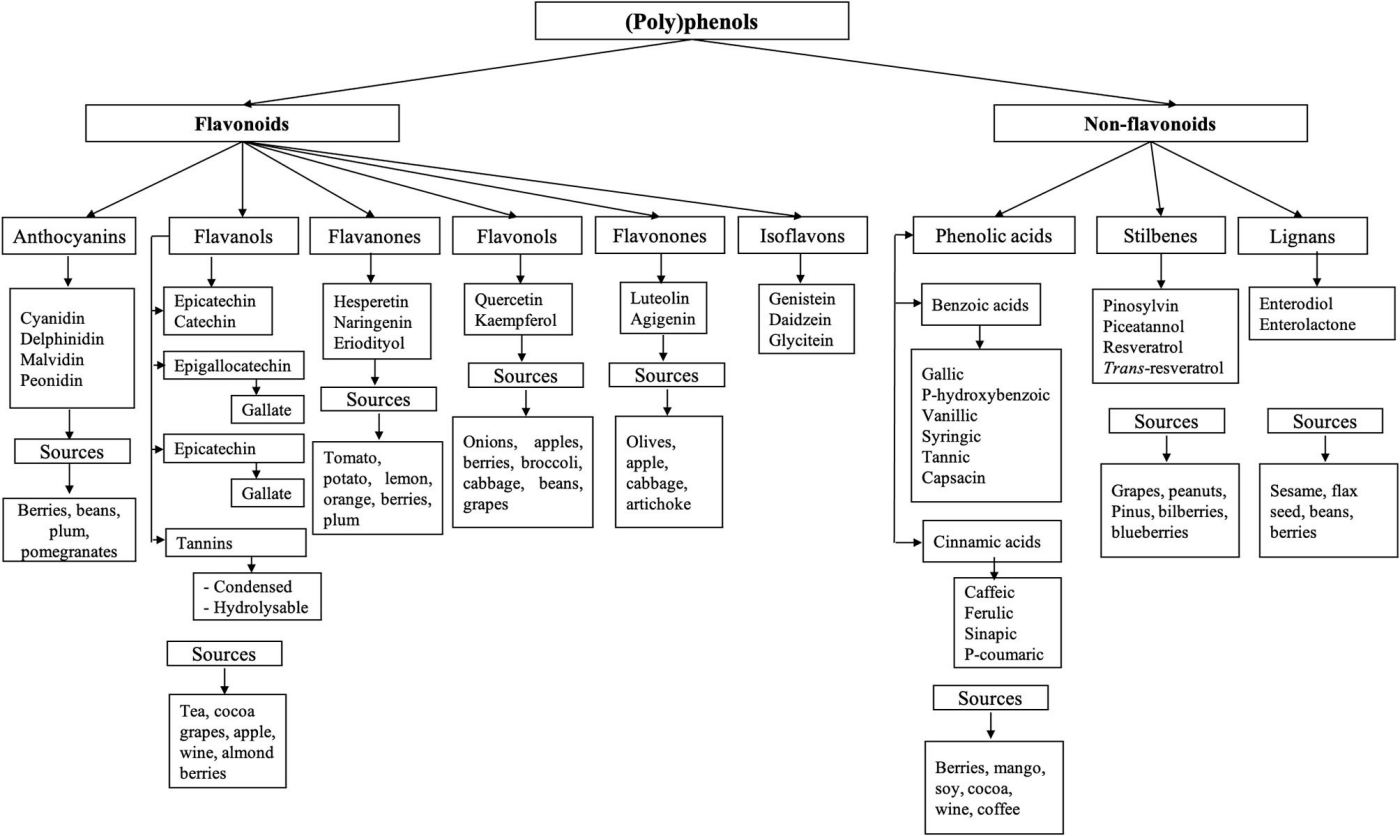
Classification of phenolic compounds. (Poly)phenols are classified into two main groups: flavonoids and non-flavonoids (62). Flavonoids are subclassified as anthocyanins, flavanol, flavanones, flavanols, flavonones, and isoflavones (62, 63). Non-flavonoid compounds include phenolic acids, stilbenes, and lignans.

(Poly)phenols are composed of an aromatic ring with at least one hydroxyl group; this structure can vary from monomers to complex polymers of high molecular weight. They are classified into two main groups: flavonoids and non-flavonoids (62). Their diversity and classification are illustrated in Figure 2. Only a small portion of (poly)phenols consumed are absorbed (5–10%), and a large proportion (90–95%), typically flavonoid aglycones and polymers, reach the colon (13, 14), where they interact with the microbiota and can exert their antimicrobial and prebiotic effects.

Prebiotics are usually confined to specific non-digestible carbohydrates (i.e., inulin, fructooligosaccharides FOS, galactooligosaccharides GOS). However, this concept was revisited recently by the International Scientific Association for Probiotics and Prebiotics (ISAPP) (12), which pointed out that plant (poly)phenols can also be considered prebiotics. Indeed, based on the recent evidence of (poly)phenol trophic utilization by gut bacteria and their promoting action on species with health benefits to the host, several (poly)phenols may fit this category. Through these prebiotic-effects, (poly)phenol-rich foods can attenuate metabolic and inflammatory diseases (14), increase host intestinal mucus production (64, 65), induce the secretion of gut antimicrobial peptides (66, 67), modulate hepatic bile acids (68, 69), and gut immunoglobulins secretion (70).

**Figure 3:**
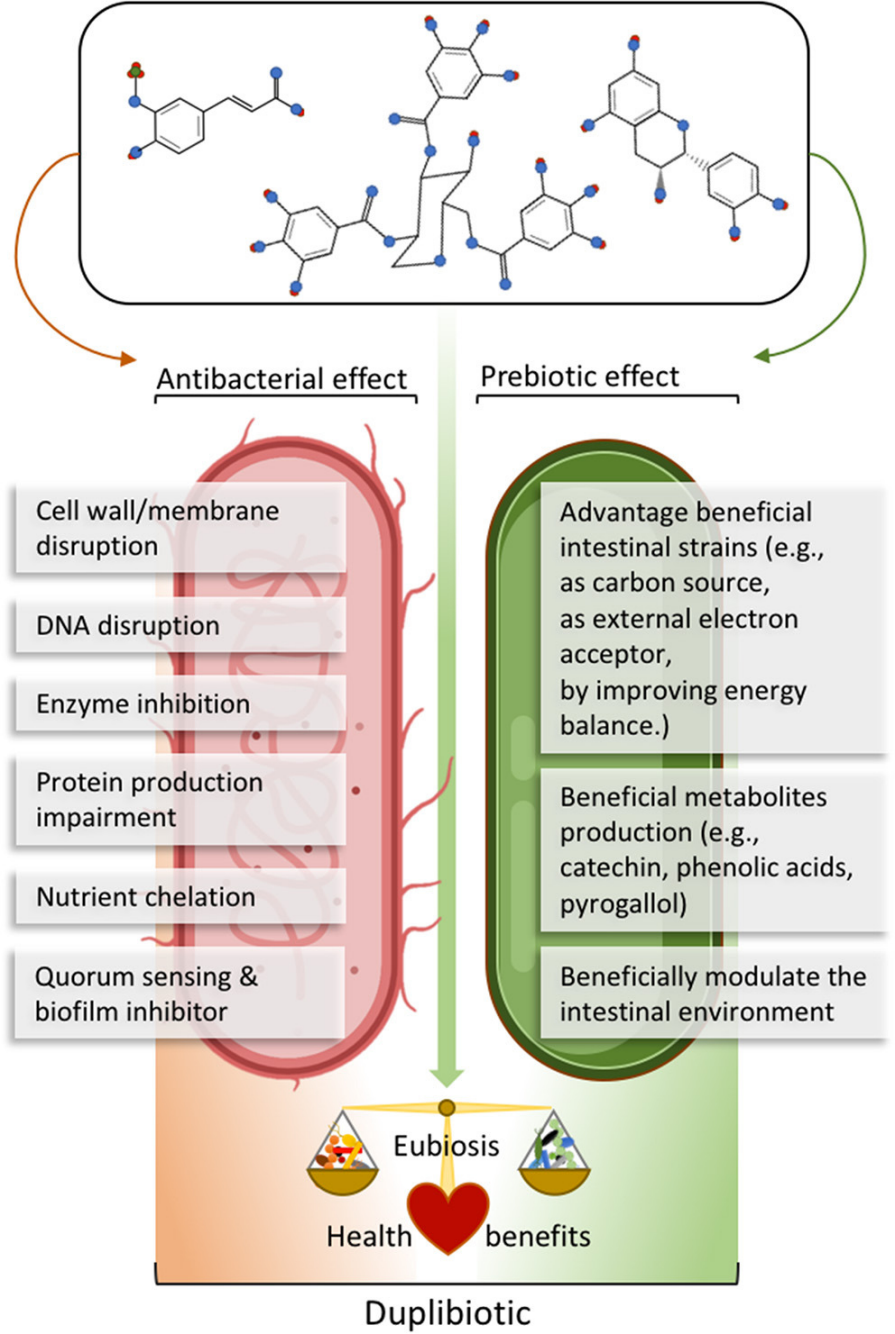
Description of (poly)phenols direct antibacterial and prebiotic effects. The antibacterial effect corresponds to the direct detrimental interaction between (poly)phenols or their metabolites and bacteria, while the prebiotic effect corresponds to the beneficial effect generated through direct bacterial metabolic utilization of (poly)phenols or their metabolites. The term *duplibiotic* design a non-digestible compound that, once reaching the colon, interacts with the gut microbiota through a dual antibacterial and prebiotic effect, favoring a eubiotic intestinal state and providing health benefits to the host.

The action of (poly)phenols on the gut microbiota often relies on dual antimicrobial and growth-stimulating (thus potentially prebiotics) effects. For example, red wine polyphenols increased the fecal abundance of Bifidobacteria, Lactobacilli, and *F. prausnitzii*, while inhibiting LPS producers, such as *E. coli* and *Enterobacter cloacae* in metabolic syndrome patients (16). In this sense, we propose the neologism *duplibiotic* to depict those inseparable substrate-induced modes of action in the gut, that is to promote beneficial bacterial species through concomitant antimicrobial and prebiotic effects (Figure 3). This term allows a more accurate and complete description of the dual effects of certain (poly)phenols.

### (Poly)phenol's Antimicrobial Mechanisms

Studies integrating multi-omic approaches have documented the pleiotropic antimicrobial mechanisms by which (poly)phenols modulate gut microbial communities (14, 71–75). For instance, (poly)phenols can interact with bacterial proteins to inhibit bacterial nucleic acid synthesis, alter cell membrane function and fluidity, modify cell wall integrity and synthesis, affect cell metabolism, and prevent biofilm formation (76). Likewise, (poly)phenols can inhibit quorum sensing (77, 78) and chelate essential metals like iron, copper, and zinc, which are essential to bacteria metabolism (79, 80). Through their antimicrobial actions, (poly)phenols can act as opportunistic pathogens inhibitors (73), protect the intestinal epithelium, and restore the microbiota homeostasis altered in several diseases.

(Poly)phenols antimicrobial action influences multiple microbial genetic responses, ranging from antibiotic resistance, metabolic responses, cell surface architecture, and stress response pathways (81). For instance, Firrman et al. (82) evaluated the growth patterns, cell morphology, and the genetic expression profiles of gut commensal bacteria exposed to different quercetin concentrations for 24 h and observed that it did not affect the growth of *Ruminococcus gauvreauii*, slightly repressed *Bifidobacterium catenulatum*, but inhibited the growth of *Enterococcus caccae*. The molecular analysis of the bacteria response to quercetin revealed putative mechanisms of tolerance/resistance. Interestingly, an increased expression of ABC transporters, a system associated with antibiotic resistance, was uncovered in *R. gauvreauii* and *B. catenulatum*. Particularly, these efflux pumps can expel harmful molecules out of the bacteria cell. In another study, a cranberry (poly)phenol extract down-regulated genes encoding for outer membrane proteins in *Escherichia coli* O157:H7 (83). Cranberry pomace and the flavonol-rich fraction had bactericidal effects against *Salmonella* strains (84); it reduced the expression of virulence genes and cell wall/membrane biogenesis after 20 h of incubation. Furthermore, (poly)phenols can also affect bacterial quorum sensing. In this case, cranberry proanthocyanins (PAC) reduced the production of QS-regulated virulence genes in *Pseudomonas aeruginosa* in a *Drosophila melanogaster* model (77, 84). Moreover, cranberry PAC polymers potentialized the action of conventional antibiotics by acting as quorum sensing inhibitors in bacteria (85, 86).

Metal ion-chelating properties of (poly)phenols can efficiently reduce pathogen colonization in the gut by decreasing the activity of metalloenzymes and by interfering with oxidative phosphorylation necessary for cytochrome's heme production (79). It has long been known that iron availability is crucial for bacterial growth, and iron sequestration can affect pathogens' growth and metabolism. For instance, iron-chelating gallotannins from mango inhibited Gram-positive food pathogens and Gram-negative *E. coli*, whereas they had no or little effects on lactic acid bacteria, as this last group was not heme dependent. Adding iron in the growing media decreased the heme-dependent inhibitory impact of polyphenols on bacteria (80).

In the context of obesity, the antimicrobial properties of (poly)phenols have been linked to the prevention of gut dysbiosis. Many gut opportunistic bacteria encountered in cases of obesity and IBD are inhibited by (poly)phenol-enriched diets (Table 1). In line with this, a black raspberry phenolic extract inhibited 10 bacterial genera from the phylum *Firmicutes*, and especially *Ruminococcus*, while the abundance of *A. muciniphila* was increased 157-fold in C57BL/6J mice fed an obesogenic diet (120). Also, a 4-week oolong tea (poly)phenols treatment repressed gut dysbiosis in HFD-induced obesity mouse model colonized with human gut microbiota; it increased the taxa of *Prevotellaceae*, and reduced *Ruminococcaceae, Lachnospiraceae* and, *Veillonellaceae* (74). The dietary administration of epigallocatechin-3-gallate (EGCG) to C57BL/6N mice fed an HFD, significantly lowered the abundance of the genera *Mucispirillum, Ruminococcus, Lachnospiraceae, Desulfovibrionaceae* and, *Anaerotruncus*, and significantly increased the abundance of the genera *Adlercreutzia, Akkermansia, Allobaculum*, and *Parabacteroides* (121). Those changes were associated with improvement in bile acid dysregulation (121).

**Table 1:** *Polybiotic* nature of selected (poly)phenols.

**Polyphenol sources**	**Study model**	**Promoted symbionts/associated taxa**	**Inhibited potentially pathogenic bacteria**	**Polyphenol-gut transforming bacteria**	**Beneficial effects**	**References**
Catechins	*In vitro*	*Lacticaseibacillus rhamnosus*, *Lactobacillus acidophilus*.	*Escherichia coli*, *Listeria monocytogenes*, *Salmonella enterica* subsp. *enterica* serovar Typhimurium.	*Adlercreutzia equolifaciens* subsp. *celatus*, *Adlercreutzia equolifaciens* subsp. *equolifaciens*, *Eggerthella lenta*, *Lacticaseibacillus casei* 01, *Lacticaseibacillus plantarum* IFPL935^*^, *Slackia equolifaciens*, *Slackia isoflavoniconvertens*.		(87–91)
	Animal	Akkermansia, Lactobacillus^**^.			Attenuated serum alanine aminotransferase and serum endotoxin levels in non-alcoholic induced steatohepatitis.	(92)
Ellagic acid	*In vitro*		*Bacteroides fragilis*, *Clostridium perfringens*, *Enterocloster clostridioformis*, *Erysipelatoclostridium ramosum*.	*Bifidobacterium* pseudocatenulatum INIA P815^*^, *Ellagibacter isourolithinifaciens*, *Gordonibacter pamelaeae*, *Gordonibacter urolithinfaciens*.		(93–96)
	Animal				Cancer chemoprevention in a buccal pouch hamster carcinogenesis model. Antioxidant effects and protection against cisplatin-induced nephrotoxicity in rats.	(97, 98)
Ferulic acid	*In vitro*		*E. coli*, *S*. Typhimurium.	*Bifidobacterium longum*, *L. plantarum*, *L. acidophilus*, *Lactobacillus helveticus*, *Lactobacillus johnsonni*, *Limosilactobacillus*, *reuteri*, *Limosilactobacillus*, *fermentum*.		(90, 99–101)
	Animal	*Lactobacillus, Parabacteroides*.			Improve cardiac function in mice with transverse aortic constriction.	(102)
Gallic acid	*In vitro*	*L. acidophilus*, *L. rhamnosus*.	*E. coli*, *S*. Typhimurium.	*L. plantarum* group, Lactobacillus gasseri^*^, F191, JCM 5343.		(90, 103)
	Animal	*Bacteriodes*	*Prevotella*		Improved glucose and insulin homeostasis in an ulcerative colitis model. Increased thermogenesis. Reduced body gain (without food intake changes).	(104–107)
Gallotannins	*In vitro*		*Bacillus cereus*, *Clostridium botulinum*, *Campylobacter jejuni*, *E. coli*, *L. monocytogenes*, *Staphylococcus aureus*, *S*. Typhimurium.	L. plantarum^*^ ATCC 14917.		(90, 103)
	Human	*Lactococcus lactis*	*Clostridium leptum*		Decreased plasma endotoxins. Improved plasma pro-inflammatory cytokines and metabolic hormones in obese subjects.	(108)
Hesperidin	*In vitro*	*Lactobacillus* sp. DSM 20059, *Bifidobacterium bifidum* NCFB 2235^***^.	*Enterococcus faecalis*, *E. coli*, *Salmonella enterica subsp. enterica* serovar Typhi, *S. aureus*, *Staphylococcus epidermidis*, *Pseudomonas aeruginosa*.	*Bifidobacterium animalis ssp. lactis* BB12, Bifidobacterium breve^*^ WC 0422, *Bifidobacterium catenulatum* ATCC 2753, B. pseudocatenulatum^*^ WC 0400, WC 0401, WC 0403, WC 0407, WC 0408 *Lacticaseibacillus paracasei*, *Levilactobacillus brevis* ATCC 367, *L. acidophilus* LA-5.		(109–113)
	*Animal*	*Bacteroidaceae* *Bifidobacterium* *Lactobacillus*/*Enterococcus group*			Attenuation of intestinal inflammation, antioxidant protection, and improvement of intestinal permeability in a mouse model of dextran sulfate sodium-induced colitis. Improved lipid metabolism in a rat model of diet-induced obesity.	(114–117)
Quercetin	*In vitro*	*B. bifidum* NCFB 2235^***^, *Lactobacillus* sp. DSM 20059.	*E. coli*	*B. fragilis*, *C. perfringens*, *E. coli*, *Eubacterium ramulus*, *Flavonifractor plautii*, *L. acidophilus*, *Weissella confusa*.		(109, 112, 113, 118)
	Animal	Akkermansia^***^, Bacteroidetes^**^.	Helicobacter^**^, Proteobacteria^**^.		Decreased insulin resistance, reduced intrahepatic lipid accumulation, and restored intestinal barrier in mice with diet-induced non-alcoholic fatty liver disease.	(119)

* *Strain-specific metabolization, some or most strains belonging to these species do not present this ability.*

** *In the context of a high-fat diet*.

*** *Observed trend*.

### (Poly)phenol's Prebiotic Action

(Poly)phenol-rich extracts exert a stimulatory effect on bacteria with crucial biological roles, such as probiotic species belonging to Lactobacilli and Bifidobacteria genera (32, 122, 123). This stimulation stems from either a (poly)phenol-induced shift in the microbial ecological niches, the reestablishment of the mucosal pro- and anti-inflammatory balance, the inhibition of potentially pathogenic bacteria, or from the direct utilization of (poly)phenols by gut bacteria. In this sense, it is important to distinguish the true prebiotic effect from indirect prebiotic-like effects induced by (poly)phenols. According to the most recent definition of prebiotics provided by the ISAPP, a compound will only be considered a prebiotic if its benefits to the host are brought about by its selective utilization by members of the gut microbiota (12). This criterion is of paramount importance to the concept of prebiotic as it excludes from its scope, the bacteriostatic, antimicrobial, and antibiotic compounds, as well as certain minerals and vitamins, even though they might beneficially impact the structure and activity of the microbiota, and thus contribute to host health. It is essential to evaluate the selective utilization of (poly)phenols by the gut microbiota since their antimicrobial activity can be improperly interpreted as a prebiotic effect. Indeed, the ISAPP experts highlighted that beneficial effects on the oral microbiota induced by cocoa and cranberry's polyphenols extracts (124, 125) are not be considered a prebiotic effect since it relies on bacteriostatic effects on susceptible pathogenic bacteria rather than a direct stimulatory effect on beneficial ones (12). Moreover, for similar reasons, bacterial (poly)phenol degradation without utilization—as in a detoxification process, for instance—cannot be considered a genuine prebiotic effect even if it leads to the liberation of metabolites able to produce beneficial effects or to participate in a trophic network. To illustrate this situation, quercetin degradation into dihydroxyphenylacetic acid by *Eubacterium ramulus*, a detoxification process protecting sugar transporters from inactivation (11), cannot be considered a prebiotic effect. In the same way, the bloom of beneficial bacteria resulting from a reduced microbial competition caused by (poly)phenol inhibitory action on co-exclusive opportunistic microbes would not be considered a prebiotic effect, but an indirect stimulation. Such a case is observed with the bloom of *A. muciniphila*, often prompted by (poly)phenols. However, as of now, its (poly)phenol-degrading ability has not been established

Several *in vitro* studies have demonstrated the direct positive impact of (poly)phenols on beneficial bacteria in pure culture (Table 1). Particularly, numerous strains from the genus *Lacticaseibacillus, Lactiplantibacillus*, and *Lactobacillus* grow faster when cultured with purified (poly)phenols. Furthermore, Bifidobacteria strains are also promoted by (poly)phenols *in vitro*. Both Lactobacilli (126) and Bifidobacteria have proven effects on health, and several species are recognized as probiotics with known modes of action (127–129). Certain modes of action by which (poly)phenols promote bacterial growth have been elucidated. (Poly)phenols provide carbon sources (after microbial deglycosylation), act as electronic acceptors (as is the case with hydroxycinnamic acids) or generate proton motive forces during their metabolization (as is the case with gallic acid) (Figure 3). (Poly)phenols also promote the growth of *Bacteroides* spp. *in vivo* (Table 1). The bloom of this genus has been related to its capacity to use (poly)phenols as a trophic substrate (130, 131). *B. thetaiotaomicron*, a bacterial keystone from this genus, is recognized as a next-generation probiotic (32) with carbohydrate-catabolic abilities. Among the bacterial species prompted by (poly)phenols, a bacterial consortium expressing PAZymes often stands out accompanied with co-occurring beneficial taxa after (poly)phenols intake (Table 1). However, there is still a paucity of results on the induction of PAZymes in several gut bacterial species promoted by dietary (poly)phenols.

## PAZymes and Gut Microbial Species

The prebiotic action of (poly)phenols on the gut microbiota may directly stem from the activation of PAZymes like tannase, quercetinase (family of cupin-type dioxygenases: flavonol 2,4-dioxygenase, quercetin 2,3-dioxygenases), gallate decarboxylase, esterase, and phenolic acid decarboxylase enzymes (132–138), leading to both the generation of bioaccessible phenolic metabolites and microbial cross-feeding interactions in the gut. (Poly)phenol-rich powders and high molecular-weight polymeric fractions significantly increased the proportion of *Eggerthellaceae* and *Coriobacteriaceae* families (i.e., *Adlercreutzia equolifaciens*) in the gut microbiota of humans and animals (19, 72, 122, 139), two families displaying unique ability to break down (poly)phenols and transform them into trophic growth factors. Among the species belonging to these families, *Gordonibacter* spp., *Eggerthella lenta*, and *A. equolifaciens* have been identified as (poly)phenols-degrading gut bacteria (140). These species are known to metabolize ellagic acid and flavan-3-ols and have the capacity to cleave the C-ring of all (epi)catechin stereoisomers and other derivatives (141). Similarly, the growth-promoting activity of blueberry (poly)phenols on Bifidobacteria and Lactobacilli strains has been linked to their (poly)phenol-metabolizing abilities (132, 134–136, 142). Finally, Lactobacilli species, such as *L. plantarum, Lacticaseibacillus casei*, and *Lactobacillus acidophilus* thrive on (poly)phenolic-enriched media owing to their ability to metabolize tannins (27, 134, 143–145).

To date, the (poly)phenol metabolism of *L. plantarum* is one of the best-studied among probiotic bacteria. This ubiquitous microorganism colonizes several (poly)phenol-rich niches, such as plants phyllosphere and fermented vegetables, as well as dairy foods and the human gut. *L. plantarum* pan-genome includes several genes encoding enzymatic functions involved in the utilization of (poly)phenols (146, 147). Specifically, these include intracellular tannase (*tanB*_*LP*_), gallate decarboxylase (*lpdB, lpdC*), aryl glycosidase (10), rhamnosidases (*rhaB1, rhaB2*) (148), phenolic acid decarboxylase (*hcrB, lp_3665*), and vinylphenol reductase (*lp_3125*). Other genes are, however, highly strain-dependent: extracellular tannase (*tanA*_*LP*_), a broad esterase (*est_1092*, with tannase and feruloyl esterase activity), and a non-identified enzyme(s) able to produce diphenylpropan-2-ol from catechins (87, 149). When exposed to plant environments, *L. plantarum* modifies its core metabolic pathways to save energy and utilizes alternative NAD^+^ generation routes (150). Interestingly, *L. plantarum* transcriptomic response to tannins (as well as tannic and gallic acid) involves the overexpression of genes related to its gastrointestinal survival (*lp_2940* [cell surface protein] with gallic acid, but also *argG* [argininosuccinate synthase], *copA* [copper transporting ATPase], *napA3* [Na(+)/H(+) antiporter] with tannic acid), indicating that this microorganism may be highly adapted to survive in the context of a tannin-rich diet (143). These metabolic characteristics make *L. plantarum* a prime candidate for the development of synbiotics aiming to potentialize the benefits of (poly)phenol-rich diets. The prebiotic potential of (poly)phenols thus appears dependent on the arsenal of PAZymes triggered by probiotics and commensal microbes.

### Transformation of Gallic Acid Esters and Polymers

#### Tannases

Tannases or tannin acyl hydrolases represent a large esterase enzyme family presenting a broad range of substrates specificities that vary according to the enzyme molecular structure (133). Bacterial tannases can hydrolyze gallate and protocatechuate esters found in tannins and other phenolic compounds. However, it is unclear if gut bacterial tannases hydrolyze tannins with higher chemical complexity, such as ellagitannins (43).

Tannase activity has been mainly reported in lactic acid bacteria such as *L. plantarum* (151), *Lactiplantibacillus pentosus, Lactiplantibacillus paraplantarum, L. acidophilus, Pediococcus acidilactici*, and *Pediococcus pentosaceus* (152). The presence of this enzyme has also been noted in other gut bacteria like *Roseburia intestinalis* XB6B4 and *Slackia heliotrinireducens* (153). Gut microbial intracellular tannases may contribute to the transformation of compounds that potentially enter the microbial cell (e.g., methyl gallate, epigallocatechin gallate, and epicatechin gallate). Bacteria extracellular tannases may also transform larger molecules, such as gallotannins and, possibly, galloylated ellagitannins (154). In the colon, these gut-microbial extracellular tannases may play a key role in the prebiotic potential of tannins, releasing more bioaccessible molecules from non-absorbable polyphenols. Barnes et al. (155) found that lean individuals have higher microbial intestinal tannase activity than individuals with obesity, suggesting that gut microbial composition is likely to influence this enzyme activity. Gut tannases appear to stimulate the production of gallotannin-colonic metabolites, as higher concentrations of these metabolites (i.e., 4-*O*-methylgallic acid, 4-*O*-methylgallic acid-3-*O*-sulfate, and pyrogallol-*O*-sulfate metabolites) are later found in the systemic circulation of lean individuals eating a gallotannin-rich diet (400 g of mango pulp). Interestingly, subjects receiving an enriched gallotannin diet for six weeks tended to increase their tannase activity (155), which indicates that dietary strategies may reestablish this gut enzymatic function.

#### Gallate Decarboxylases

Gallate decarboxylases are microbial enzymes that transform gallic acid into pyrogallol. These PAZymes are highly present in lactic acid bacteria isolated from plant sources, including those belonging to the *L. plantarum* group and some *Lactobacillus gasseri* (103). Noteworthy, gallate decarboxylase favors energy uptake in *L. plantarum*, by generating a proton motive force during gallic acid metabolism (27). In the host, gallate decarboxylase-producing probiotic bacteria may enlarge the metabolic benefits induced by gallic acid and gallic acid-rich (poly)phenols. Fang et al. (51) observed that a gallate decarboxylase-producing *L. plantarum* strain (WCFS1), concomitantly administered with gallotannins (gallic acid glycosidic polymers), tended to potentialize the gallotannins antiobesity effects in a gnotobiotic mice model. The brown adipose tissue of *L. plantarum*-supplemented mice had higher mRNA levels of thermogenic genes when compared to mice receiving only gallotannins. Bacteria producing decarboxylase may thus boost pyrogallol production in the gut (51), entailing potential antiobesity and anticarcinogenic effects (156, 157). A high-throughput sequencing approach revealed that this enzymatic function is also present in different phylogenetic divisions of the gut microbiota. Gallate decarboxylase was predicted in strains of *Erysipelotrichaceae* bacterium, *Enterobacter cloacae, Actinomyces glycerinitolerans, Anaerostipes hadrus, Enterococcus raffinosus, Pediococcus ethanolidurans, Klebsiella michiganensis, Blautia* sp., *Dorea longicatena, Clostridium butyricum* (158).

### Transformation of Hydroxycinnamic Acid Esters

#### Feruloyl Esterases

Feruloyl esterases (also called hydroxycinnamoyl esterases) are a large family of PAZymes presenting different hydrolyzing capacities toward hydroxycinnamic acid esters (ferulic, p-coumaric, caffeic, sinapinic, or diferulic acids esters) (159), frequently found in plant cell walls, as in wheat bran (160), berries (161), and Jerusalem artichoke (162). Because they are usually covalently bound to fibers, they are not readily bioaccessible. Therefore, feruloyl esterases can improve the bioavailability of these functional compounds. This enzymatic activity has been reported so far in *Bifidobacterium longum* (99)*, Lactobacillus helveticus, Lactobacillus johnsonni, Limosilactobacillus reuteri, Lactobacillus acidophilus, Limosilactobacillus fermentum* (100), and *L. plantarum* (163). However, substrate affinity may greatly differ between bacterial esterases. For example, *L. fermentum*'s feruloyl esterase has a greater hydrolytic activity on methyl caffeate, than on trans *p*-coumarate or methyl ferulate (164). Feruloyl esterase-producing lactic acid bacteria have a broad spectrum of applications as they increase the bioaccessibility of ferulic and other hydroxycinnamic acids in fermented foods, such as fermented whole grain barley and oat groat (165). Additionally, supplementation with feruloyl esterase-producing probiotic strains, such as *L. fermentum* CRL1446, can greatly improve intestinal esterase activity and oxidative stress parameters of supplemented mice (166).

In humans, hydroxycinnamoyl esters hydrolysis first takes place in the small intestine, mainly by mucosal esterases (167). However, in many food sources, such as bran and aleurone, ferulic acid is mostly covalently bound to arabinoxylans and other cell wall polysaccharides able to resist digestion in the upper gastrointestinal tract (168). Once in the colon, the resident microbiota cooperatively breaks the cell wall polysaccharides and release ferulic acid from its chemical bonds. Using *in vitro* fermentation, Duncan et al. (169) pinpointed the role of butyrate-producing gut microbiota species, such as *Bacteroidoita* phylum, and their capacity to release ferulic acid from wheat bran fermentation. *Bacteroidota* species are primary degraders of arabinoxylan in the colon, deploying a differential enzymatic machinery according to the arabinoxylan type (soluble vs. complexed with ferulic acid side chains) (170). The genera of *Bacteroides, Eubacterium, Roseburia*, and *Butyrivibrio* (*Lachnospiraceae*) degrade xylans, while the genera of *Ruminococcus* and *Faecalibacterium* can degrade hemicelluloses or pectin. In line with this, *Ruminococcus* spp. and *F. prausnitzii* were prompted by wheat bran (arabinoxylan-rich phenolic acid-rich polysaccharides), resulting in the release of ferulic acid in the human gut microbiota (171). This phenolic compound was then rapidly reduced to produce phenylpropionic acids, known to benefit host health. Overall, many types of interactions between polysaccharide and feruloyl-esterase-producing bacteria may occur within the gut microbial ecology, potentially enriching the microbial diversity and metabolomes, including SCFA and phenolic metabolites. Ferulic acid is a highly bioavailable metabolite (172) that inhibits platelet activation (173), modulates insulin signaling (174), and reduces metabolic syndrome symptoms in diabetic rats (175). It mimics many of the beneficial effects of other (poly)phenols on cancer, cardiovascular, and cognitive diseases, among others (176).

#### Phenolic Acid Reductase

Phenolic acid reductases are enzymes able to reduce hydroxycinnamic acids, and produce substituted phenylpropionic acids [phloretic, 3-(3-hydroxyphenyl) propionic acid (3-HPPA), dihydrocaffeic, and dihydroferulic acids]. These colonic microbial metabolites have been found in the urine and plasma of subjects following a supplementation of a green and roasted coffee blend as hydroxycinnamic acids source (177). Phenolic acid reductase activity has been reported in *L. fermentum, Latilactobacillus curvatus. L. reuteri, Furfurilactobacillus rossiae*, and *L. plantarum* (178). Interestingly, these PAZymes confers an energetic advantage for strict heterofermentative lactic acid bacteria, as they can use these phenolic acids as external electron acceptors in the pentose-phosphate pathway (28). The resulting (poly)phenol-derived metabolites have higher antiplatelet properties than the parental compounds (173). They also prevent monocyte endothelial adhesion (179) and may protect against pancreatic-β-cell dysfunction *in vitro*.

### Transformation of Phenolic Glycosides

#### α-L-rhamnosidases

The α-L-rhamnosidases are enzymes able to release aglycones and glucose by cleaving the terminal α-L-rhamnoses present in glycosylated phenolic molecules (180). Since the human intestine possesses no rhamnosidase activity (168, 181), the vast majority of these rhamnose glycosides remain unabsorbed and reach the colon. The ability to transform specific rhamnose glycosides frequently found in citrus fruits, such as hesperidin and rutin, has been demonstrated in some probiotic bacteria. Hesperidin glycosides are converted, among others, by strains of *Levilactobacillus brevis, Lacticaseibacillus paracasei, L. acidophilus* (170), *Bifidobacterium animalis, Bifidobacterium breve, B. catenulatum* and *Bifidobacterium pseudocatenulatum* (171). However, the hydrolyzing capacity of these species is highly strain-dependent and varies according to the structure of the rhamnose glycoside. For instance, the rutin structure is less accessible to bacterial rhamnosidases, and only certain probiotics, such as *L. fermentum*, can degrade it to a small extent (109, 170). In addition, rhamnosidase activity may be influenced by certain carbon sources and other phenolics present in the media. Particularly, bacterial α-L-rhamnosidases were stimulated by narcissin and rhamnose (170) while inhibited by glucose (148). Other rhamnosidase-producing bacteria belonging to *Lachnospiraceae, Enterobacteriaceae, Tannerellaceae*, and *Erysipelotricaceae*, may be involved in the transformation of rhamnose glycosides, such as dietary rutin (170).

#### β-glucosidases

β-glucosidases are heterogeneous and widespread enzymes that enable bacteria to obtain carbon sources from glucosides catabolism (182). (Poly)phenol glucosides, occurring primarily as *O*-glucosides (OH-coupled), are deglycosylated by β-glucosidases, resulting in the release of sugars and aglycones: a free form in which (poly)phenols may passively diffuse across tissues (183, 184). C-Glycosides (C-C-coupled) are less frequent in nature and more difficult to degrade. Details of C-glycoside cleavage by intestinal bacteria have been recently reviewed by Wei et al. (185). β-glucosidases are produced in response to glucoside-rich environments by several intestinal bacteria such as *B. thetaiotaomicron, Bifidobacterium spp., Blautia producta, Erysipelatoclostridium ramosum, E. coli*, and *L. plantarum* (10, 186, 187). Zyzelewicz et al. (188) recently reported an increase in cecal β-glucosidase activity in high-fat-fed rats after a cocoa bean extract supplementation, indicating that diet composition may modulate this enzymatic activity. However, it is not clear if this (poly)phenol-induced activity stems from microbiota compositional changes or from alterations of the microbiome function, as only enzymatic activity was assessed.

Many lactic acid bacteria (*Streptococcus thermophilus, L. acidophilus, Lactobacillus delbrueckii ssp. bulgaricus*) are also able to produce β-glucosidases (182). Because of their safety, these β-glucosidase-producing-bacteria are used to increase the bioaccessibility of phenolic glucosides during food fermentation (189). One example is the hydrolysis of the glucoside daidzin, contained in soybeans, into its aglycone daidzein. This aglycone can be further converted into equol, a potent bioactive compound, by four subsequent enzymes (daidzein reductase, dihydrodaidzein reductase, tetrahydrodaidzein reductase, and dihydrodaidzein racemase). In addition to its anticancer, cardioprotective, and neuroprotective activities, equol has gained attention for its capacity to modulate estrogen levels, reducing menopause symptoms (190). Intestinal (poly)phenol-degrading bacteria such as *Eggerthella* spp. and certain strains of *Adlercreutzia equolifaciens* (141, 191, 192) produce equol from daidzein aglycone. This enzymatic machinery is also present in probiotic species such as *B. breve, B. longum, Lactococcus garvieae, and Lactobacillus intestinalis* (189). These observations have fostered the emergence of probiotic strategies and have resulted in better clinical outcomes in equol non-producers (193).

## (Poly)phenol-Derived Beneficial Metabolites

Microbial phenolic metabolites, like their native compounds, may exert antioxidant, antiproliferative, and anti-inflammatory activities (194, 195). The production of these beneficial metabolites is strongly dependent on the PAZyme production by the gut microbiota of each individual, a phenotype so-called metabotype. This important concept has been recently and thoughtfully reviewed elsewhere (43). Indeed, the notion of different metabotypes would explain, in part, the interindividual differences in the health outcomes observed after (poly)phenol intake. Therefore, the presence of (poly)phenol-degrading bacteria in the gut microbiota is crucial to potentiate the bioactivity of parent (poly)phenols. This is the case of bacteria producing equol from daidzin, enterolactones from lignans, 8-prenyl naringenin from xanthohumol, and urolithins from ellagitannins (196).

It is also believed that PAC bioactivity can be potentiated by gut bacteria to produce active isomers of valerolactones. Phenyl-γ-valerolactones and phenylvaleric acid derivatives, 5-(3′,4′-dihydroxyphenyl)-γ-valerolactone, and 4-hydroxy-5-(3′,4′-dihydroxyphenyl)-valeric acids are flavan-3-ols derived metabolites from fecal fermentation of PACs. Using Orthogonal Projections to Latent Structures Discriminant Analysis (OPLS-DA) and Variable Importance in Projection (VIP) analysis, Rocchetti et al. (197) reported fermentation markers linking the chemical structure of (poly)phenols to the colonic pathways in which they are involved. In that study, changes in the phenolic profiles after *in vitro* fermentation of (poly)phenol-rich nuts (high in PACs) were evaluated by both untargeted UHPLC-QTOF and targeted UHPLC-Orbitrap mass spectrometry. The OPLS-DA model revealed catabolic pathways involving PAC C-ring fission of the flavan-3-ol-backbone, leading to the formation of 5-(3′,4′-dihydroxyphenyl)-γ-valerolactones. Subsequent oxidation reactions are thought to produce lower-molecular-weight phenolic acids such as 3,4-dihydroxyphenylacetic acid and hydroxybenzoic acids. These reactions are driven by an enzymatic repertoire encoded by species belonging to *Coriobacteriaceae* (*A. equolifaciens*), *Lactobacillaceae* (*L. plantarum*), *Ruminococcaceae* (*Flavonifractor plautii*), and *Eggerthellaceae* (*E. lenta, Paraeggerthella, Gordonibacter* and *Slackia equolifaciens*) (140, 198, 199). The bioavailability and potential bioactivities of valerolactones are very topical and the focus of recent research (200, 201). Indeed, valerolactone isomers were recently shown to reduce monocyte adhesion in vascular endothelium, potentially preventing atherosclerosis (202). These metabolites appear to pass the blood-brain barrier and might thus be involved in neuroprotective effects of (poly)phenols-rich diets (203–205). Obviously, changes in gut microbiota functions may have repercussions on the production or degradation of certain metabolites, having marked effects on the well-being of the host. Studies have found that gut microbiota xenobiotic metabolism, particularly linked to (poly)phenols, is associated with reduced inflammation, promoting gut health in the context of obesity (206, 207).

The urolithin A, a metabolite derived from ellagitannins degradation, is considered a metabotype biomarker associated with reduced cardiometabolic risk (208, 209). It is worthy to note that urolithins also exert antimicrobial and anti-quorum sensing activities, for example, by inhibiting N-acyl homoserine lactones (AHL), a QS signal molecule involved in cell population density and biofilm formation (210). In fact, urolithins A and B can alter the expression levels of genes critically involved in the synthesis of lactones and swimming motility of the enteropathogenic *Yersinia enterocolitica* (210). Dietary derived (poly)phenols metabolites, like their parent compounds, may thus exert antimicrobial effects in the colon and suppress the colonization of opportunistic bacteria, contributing to reestablish gut dysbiosis. This inhibitory action on opportunistic bacteria, in addition to (poly)phenol-induced changes in the gut barrier and immune responses, is also a potential mechanism indirectly fostering gut bacteria with health benefits to the host.

## Prebiotic-Like Effects of (Poly)phenols: *Akkermansia* as a Typical Example

(Poly)phenols can induce ecological shifts, that is, cause important changes in the structure, the functions of microbial ecosystems leading to alternative states reconfiguration (211, 212). Indeed, (poly)phenol-induced shifts manifest themselves into enterotype-like clustering of the gut microbiota. For instance, using CCA-ordinations based on the gut microbiota composition, mice fed an obesogenic diet supplemented either with berry (poly)phenols or fibers clustered in a distinct group. In this case, the obese mice were characterized by a *Firmicutes/Ruminococcus* enterotype enriched in *Lachnospiraceae* and *Ruminococcaceae* taxa (139), which are known to be opportunistic mucosa-associated bacteria. On the contrary, the gut microbiota of mice fed with a (poly)phenol-rich cranberry powder clustered in the contrasting enterotypes of *Bacteroidetes*/*Muribaculaceae* and *Prevotella*/*Akkermansiaceae*, which were enriched in *Eggerthellaceae*, lactobacilli, *Akkermansia*, and *Lachnospiraceae*_NKA136_group (139). Besides their *duplibiotic* action, these important changes in the gut microbiota can be linked to the prebiotic-like effects of (poly)phenols in the intestinal environment (20, 213, 214).

(Poly)phenols thus have the capacity (1) to free ecological niches by hindering the potential opportunistic pathogens, (2) to re-establish the normal function of the mucosal epithelial barrier and its immunological response, and (3) to reduce oxidative agents (reactive oxygen species and free radicals) (215). These three events favor an increased abundance of beneficial gut bacteria bearing health benefits to the host, which underly their indirect prebiotic-like effect. One typical example is the case of *A. muciniphila*.

Accumulating evidence confirm the impact of dietary (poly)phenols on *A. muciniphila*. Particularly, grape (poly)phenols, apple PACs, EGCG, puerarin, A-type PAC-rich cranberry extract have been shown to dramatically increase the growth of *A. muciniphila* and decreased the proportion of *Firmicutes* to *Bacteroidetes* in cecal and fecal samples of animals, in association with an improved host metabolic phenotype (14, 16, 17, 66, 216). Likewise, (poly)phenolic compounds such as chlorogenic acid, caffeic acid, quercetin, resveratrol, trans-resveratrol, and malvidin-3-galactoside promoted *Akkermansia* in dextran sulfate sodium (DSS) induced colitis C57BL6 mice model and in mice with liver cancer (217–220). As there is no precise evidence of the PAZymes-producing ability by *A. muciniphila* to utilize the aforementioned (poly)phenol compounds, this stimulating effect should be attributed to a prebiotic-like effect rather than a direct prebiotic one.

In analogy to the well-described promoting effect of antibiotics on *Akkermansia*, (poly)phenols induced blooms might be a collateral effect of (poly)phenol's antimicrobial action, in addition to their action on the synthesis of mucin by the gut epithelium (64, 65). Indeed, this bacterium can physiologically adapt and resist broad-spectrum antibiotics, conferring it a molecular advantage to colonize the colon (221–223). *Akkermansia*'s resistance to phenolics, conjugated to a reduced competition from (poly)phenol-susceptible microbes, and the liberation of ecological niches occupied by opportunistic bacteria might explain its bloom in the presence of (poly)phenols. This outcome is illustrated, for example, by the suppression of the taxonomic abundance and activity of opportunistic bacteria colonizing the colonic mucosa such as *Ruminococcus* species (224), which in turn, reduce the microbial competitiveness and ability of mucosa-associated species to grow, as observed with *A. muciniphila*. The latter has a trophic preference to exploit the mucin glycoproteins and inhabit mucin-rich specialized niches, as the host epithelium (225), where it participates in a symbiotic crosstalk with the immune system (226, 227). Factors affecting mucin secretion and glycosylation, as well as the mucosa homeostasis thus have a major impact on the *Akkermansia* abundance. Indeed, a decrease of *A. muciniphila* in obesity and age-induced impaired mucus barrier is often observed (64, 228–231). In this sense, (poly)phenols have been shown to reinforce the mucosal epithelium by increasing the tight junction proteins (232), the mucus-secreting goblet cells, and improving the mucus thickness (19, 139), all factors favoring an optimal ecological niche for *Akkermansia* to thrive.

More studies are warranted to screen and identify the type of (poly)phenolic compounds contributing the most in prompting symbionts and modulating the functions of the gut microbiota. A recent report demonstrated a selective promoting effect of polymeric PACs rich fraction on *A. muciniphila* fecal abundance in mice fed an obesogenic diet, concomitantly with improved the colonic mucus thickness (19). Still, two questions remain unsolved; (1) does the *Akkermansia* bloom after (poly)phenols intake stems from its ability to degrade them, or (2) are the (poly)phenols boosting effects on *Akkermansia*, a consequence of the (poly)phenol-induced anti-inflammation and recovering of the intestinal mucosa-associated niche? To fill in part this gap in knowledge, we screened *in-silico* the sequenced *Akkermansia's* genome searching for genes involved in the degradation of (poly)phenols. Surprisingly, we found out that *A. muciniphila* (ATCC BAA-835) harbors two genes linked to flavonol quercetin degradation: a cupin domain-containing protein encoded by Amuc_0801 (NCBI Reference Sequence: WP_012419849.1) and γ- carboxymuconolactone decarboxylase encoded by Amuc_1806 (NCBI Reference Sequence: WP_081429203.1). The analysis of the conserved domains (CD) and protein architecture of those gene sequences led to the identification of potential matches and similarities against deposited sequences of quercetinases enzymes in the NCBI resources (Conserved domain search service). The quercetinases have been mostly described in plant-associated fungi; however, they have also been described in the gut bacterial species *Bacillus subtilis* and *E. ramulus* (104, 138, 233). Nevertheless, further in-depth characterization of the enzymatic activity coded by quercetinases-related genes in this species is required.

## Trophic Interactions Influencing Microbial Ecology Upon (Poly)phenol-Rich Food Intake

From a trophic perspective, the chemical structure and polymerization degree (DP) of (poly)phenols have a large influence on the yield of phenolic metabolites and subsequent stages of their catabolism by the gut microbiota. For instance, the human fecal fermentation of a grape seed fraction rich in flavan-3-ols monomers (70%) resulted in the generation of 3-(3,4-dihydroxyphenyl)-propionic acid, which was subsequently degraded by colonic bacteria (234). However, these phenolic metabolites yielded in higher proportion when flavan-3-ols polymers rich fraction was fermented, which was significantly linked with increased growth of *Lactobacillus/Enterococcus* taxa and the inhibition of *H. histolytica*.

(Poly)phenol polymers, which represent a large proportion of those ingested in the diet, are often intimately bound through hydrophobic and hydrogen bonds to plant cell wall matrix and therefore reach the colon in the form of (poly)phenolic fibers (235, 236). A clear case of fiber-associated (poly)phenols are polymeric flavan-3-ols, whose fermentation leads to SCFA biosynthesis while releasing the so-called “non-extractable (poly)phenols” (237, 238), thereby illustrating another example of trophic interactions in the gut environment. For instance, Mateos-Martin et al. (239) studied the fate and metabolism of grape dietary fiber rich in non-extractible PAC (14.8%), in female Sprague–Dawley rats for 24 h. The grape residues obtained after extraction with 70% acetone were rich sources of non-extractible PAC polymers. Once the colonic microbiota fermented the fibers, PAC were released and progressively depolymerized into epicatechin monomers and dimers and later metabolized into smaller units. Microbially derived PAC metabolites, such as valerolactones, phenylvaleric acids, phenylpropionic acids, phenylacetic acids, benzoic acid, and cinnamic acids, were detected only in the urine of the treated group. These fibers and (poly)phenol-derived metabolites could have an overall synergistic effect on the host. Dietary fibers and (poly)phenols have been shown to act synergistically in processes like antiradical activity and lipid oxidation *in vivo* (240), and to reduce hepatic cholesterol levels in apolipoprotein E-deficient mice with atherosclerosis (241). Another intervention study in diet-induced obese mice demonstrated the selectivity of PAC bound to cranberry fibers not only in inhibiting HFHS-induced opportunistic bacteria, but also boosting the (poly)phenol-degrading families of *Coriobacteriaceae* and *Eggerthellaceae*, as well as butyrate-producing bacteria belonging to *Lachnospiraceae NK4A136* taxon; these (poly)phenolic-fiber induced microbial changes were reflected by reduced hepatic triglycerides in obese mice. Of particular interest, such a microbial modulatory effect and host health outcome were not reproduced by the blueberry PAC-free fibers (139).

It is worthy to note that (poly)phenols can also increase the production of SCFA. While the mechanisms explaining this production are not yet fully understood, this may be mediated by the physicochemical inhibition of the mouth amylase, leading to an increased concentration of complex carbohydrates reaching the gut (242), or by an increase in anaerobic bacteria (243, 244) through the reduction of oxidative molecules in the gut environment. The fermentation of glycosylated (poly)phenols reaching the gut could also represent a non-negligible source of carbohydrates for several members of the microbiota (140), potentially constituting a (poly)phenol prebiotic pathway leading to the production of SCFA. Not only (poly)phenols can favor the production of SCFA, but the latter can also affect the absorption of (poly)phenol metabolites. As a matter of fact, Van Rymenant et al. (245) have observed an increased translocation of secondary (poly)phenol metabolite from ferulic acid and hesperidin through the epithelium, in the presence of butyrate and propionate in an *in-vitro* model of Caco-2 cells. They proposed that those SCFA would increase the production of (poly)phenol transporters (MTC1, MTC4, and various ABC transporters), and that propionate could lead to the reduction of ferulic acids to dihydroferulic acids, increasing their transport across the membrane.

(Poly)phenol and carbohydrates substrates can potentiate further cooperative interactions between PAZymes-producing bacteria and those showing less or no PAZymes activity. This was observed by Rodriguez-Castano et al. (11), who evaluated in a simple co-culture model the interactions between the gut symbiont *B. thetaiotaomicron* and *Eubacterium ramulus*. The former gut symbiont has a wide capacity to degrade complex polysaccharides but is unable to degrade the flavonoid quercetin, while the latter is a quercetin-degrading species with limited capacity to breakdown polysaccharides. Indeed, *E. ramulus* has been shown to respond to the presence of quercetin in the human diet (246). In this model, the addition of starch increased *E. ramulus* quercetin degradation activity and the ensuing production of 3,4-dihydroxyphenylacetic acid, whereas high levels of butyrate were significantly induced when co-culturing with *B. thetaiotaomicron*. In contrast, when both species were cultured individually in starch and quercetin enriched media, no significant quercetin degradation nor butyrate production were observed. Hence, the release of glucose and maltose upon the starch fermentation by *B. thetaiotaomicrom* powered, in turn, the quercetin-degrading activity of *E. ramulus*. Such trophic interactions increase the potential generation of bio-active metabolites, such as SCFAs and phenolic metabolites. Particularly, the production of quercetin-derived metabolites including 3-(3,4-dihydroxyphenyl) propionic acid, 3,4-dihydroxyphenylacetic acid, 3,4-dihydroxybenzoic acid (i.e., protocatechuic acid), and phenylacetic acid (247, 248), can exert a potential role in the improvement of an impaired glucose metabolism (249, 250).

## Studying (Poly)phenols as *Duplibiotics*

**Figure 4:**
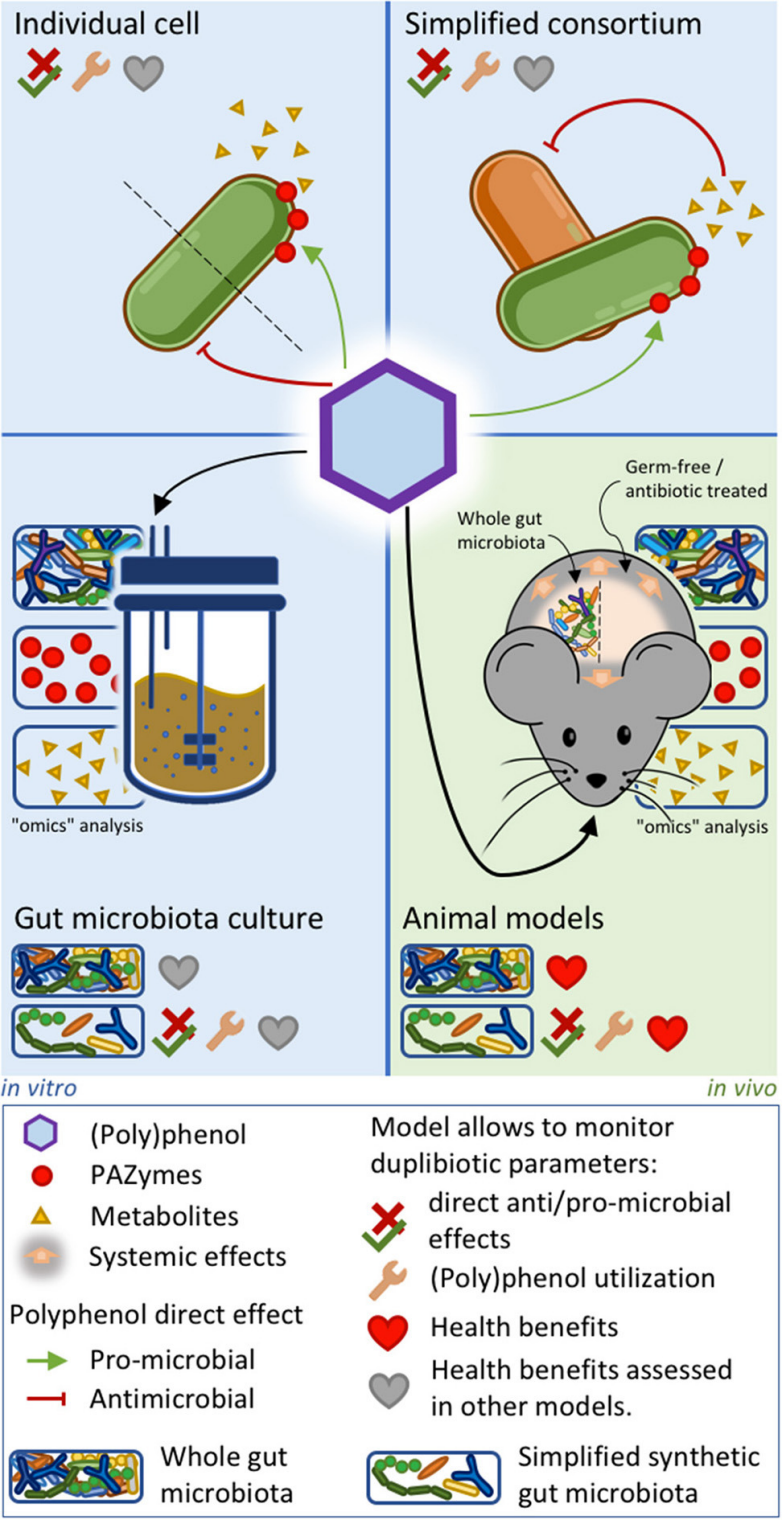
*In vitro* and animal approaches to evaluate *duplibiotic* nature of polyphenols. Individual bacterial strain or simplified bacterial consortium models allow determining (poly)phenol anti/pro-microbial effects, (poly)phenol transformation, and metabolite production. In addition, studies on simplified bacterial consortia can characterize symbiotic interactions. Gut microbiota culture and animal models combined with omics approaches provide insights on global microbial changes induced by (poly)phenols. Studies on conventionally grown, gnotobiotic, and antibiotic-treated animal models allow characterizing host health benefits. The health benefits stemming from the three *in vitro* approaches can be further demonstrated by transferring downstream metabolites into *ex vivo* systems. Synthetic microbiota inoculated in culture systems or in germ-free animals allows determining direct anti/pro-microbial effects, as well as demonstrating bacterial (poly)phenol utilization.

Deciphering the complex trophic relations taking place in the gut microbiota upon (poly)phenol-rich foods consumption is a huge challenge. Approaches involving simplified microbiotas or key microbial consortia co-cultures with different metabolic activities may help decrypt the ecological role of *duplibiotics*. Assessing the *duplibiotic* activity of a (poly)phenol requires determining both its antimicrobial activity and prebiotic effect. The simplest studies analyzing (poly)phenol effects on the gut microbiota are based on the direct challenge of individual bacterial strains by purified (poly)phenols. This approach allows identifying the metabolic activity and the associated PAZymes involved in the transformation of (poly)phenols, as well as the derived metabolites. Furthermore, (poly)phenol challenges provide information on anti/pro-microbial potential of a phenolic molecule and how bacteria resist its action (27, 132, 154, 251, 252). Studies on whole microbiota stabilized in intestinal *in vitro* systems—like the Simulator of Human Intestinal Microbiota (SHIME)—have been used to assess (poly)phenols' impact on the microbiota composition and function, to identify potential (poly)phenol-degrading bacteria and metabolites production (253). Given the complexity of the whole gut microbiota (254), co-culture experiments (11, 255) and the use of simplified synthetic gut microbial consortia combined with “omics” analyses (256, 257) may be suitable approaches to precisely assess symbiotic and trophic interactions induced by (poly)phenols. While *in vitro* models lack direct interactions with the host, they are complementary tools to elucidate mechanistic hypotheses involving the role of (poly)phenol-degrading bacterial consortia within the human gut and the downstream physiological outcomes. Indeed, transferring the metabolites-enriched supernatants (258) or the bacterial communities issued from these culture models (259) into *in-vitro* human-cell systems (253) or germ-free animals (260) can uncover the health benefits induced by (poly)phenol-rich food intake. Figure 4 summarizes the *in vitro* and animal approaches unveiling the *duplibiotic* nature of (poly)phenols. As stated by the definition of prebiotics, further well-designed and realistic human clinical studies are then essential to demonstrate the health benefits induced by the microbial utilization of (poly)phenols.

## Discussion: Toward a Comprehensive Understanding of (Poly)phenol Effects on the Microbiota and Host Health

The literature strongly supports the beneficial effect of (poly)phenol-rich foods on the gut microbiota composition, contributing to the maintenance of host energy balance and mucosal immunological responses (5, 15–17, 134, 142, 258, 261–264). In this review, we have focused on illustrating the two opposing mechanisms of (poly)phenols directly affecting the microbiota composition: the antimicrobial effect and the prebiotic effect. Not all (poly)phenols can modulate the gut microbiota through these antagonistic modes of action; however, when they do, those effects cannot be assessed separately. For this purpose, we introduce the term *duplibiotic* that describes this equivocal activity of (poly)phenols on the microbial community and expands their potential beyond a prebiotic effect *sensu stricto* by considering their whole effects that could be greater than the sum of their parts.

While the antimicrobial effect of (poly)phenols has been well-described, the prebiotic concept has commonly been misused and undistinguished from the prebiotic-like effect. Indeed, a (poly)phenol prebiotic action is often attributed after observing a stimulation of gut beneficial bacteria without confirming a utilization of the phenolic compound, as denotes the ISAPP-updated prebiotic definition. In the consensus statement on the matter (12), the ISAPP stated that the expression “a substrate that is […] utilized” were chosen for the prebiotic concept to imply “growth through nourishment.” From this perspective, we illustrated three manners by which (poly)phenols would be used by the microbiota and thus exert a genuine prebiotic effect: (1) as carbon sources, (2) as electron acceptors, and (3) as generators of a proton motive force. The first (poly)phenol purpose fits the conventional microbial utilization of well-documented carbohydrate prebiotics, but the last two extend beyond the ISAPP revisited prebiotic concept by including energy production, which is also implied in bacterial nutrition. According to those considerations, very few (poly)phenols can yet be considered as prebiotics. Other prebiotic and *duplibiotic* compounds will certainly be identified using modern omics analytical technologies, with *in vitro* synthetic approaches and clinical studies targeting the gut microbiota.

To illustrate the prebiotic potential of (poly)phenols, we described a selection of PAZymes involved in the microbial utilization of these compounds. This PAZymes abbreviation was used in this paper to regroup and discuss (poly)phenol-associated enzymes. In analogy to CAZymes, PAZymes have noteworthy similarities, as they are diverse and target different compounds, having relevance in nutrition and health. From this perspective, an initiative to group PAZymes in sequence databases, as done for CAZymes (i.e., CAZypedia, CAZyDB) (265, 266), would be of great interest. This would facilitate the identification of novel PAZymes, as well PAZymes-harboring microorganisms by coupling omics outputs, including metagenomics. Insights on PAZymes-producing species have been obtained from members of *Coribacteriaceae, Eggerthellaceae*, and *Lactobacillae*, which have the capacity to release phenolic metabolites that can impact both commensal microbiota and host health. Here, we reviewed the role of several enzymes in breaking down different (poly)phenolic classes, including the tannases, quercetinases, feruloyl esterases, gallate decarboxylases, and phenolic acid reductases that support the direct prebiotic effects of (poly)phenols on lactobacilli species and other gut symbionts. Identifying potential probiotic bacteria harboring PAZymes is of high interest since it would promote the release of bioactive phenolic metabolites and possibly induce metabotype shifts in non-producer individuals.

Even though considerable progress has been made in identifying the bacterial enzymes and genes involved in (poly)phenol metabolism, much remains to be revealed. For example, the enzymes and the genes involved in the C-ring reductive cleavage of catechin isomers and the resulting release of diphenylpropan-2-ol have not yet been discovered. *E. lenta, A. equolifaciens*, and *L. plantarum* strains were shown to be involved in these transformations (88, 89). Likewise, *F. plautii* strains can utilize diphenylpropan-2-ol metabolites (89), leading to valerolactones production. The identification of the entire set of enzymes and genes involved in the catabolism of catechin is essential to pinpoint the bacterial consortia that favor valerolactones production (267, 268). Another representative example that remains to be investigated in greater depth is the identification of urolithin-producing PAZymes and their related genes. The ability to produce urolithins from ellagic acid has been described so far in *Gordonibacter pamelaeae, Gordonibacter urolithinfaciens* (93), *Ellagibacter isourolithinifaciens* (94), and *B. pseudocatenulatum* INIA P815 (95). Particularly, the latter strain is shown to produce urolithin A, but the related enzymatic activity is apparently absent in most *B. pseudocatenulatum* strains (95). Hence, precise identification of urolithins-producing enzymes and related genes would allow the discovery of potential symbionts that could be used as therapies in subjects with non-urolithin-producing metabotypes (or low-urolithin A producers) in order to favor the release of these antiobesity (269), anti-inflammatory (270), and neuroprotective metabolites (271).

**Figure 5:**
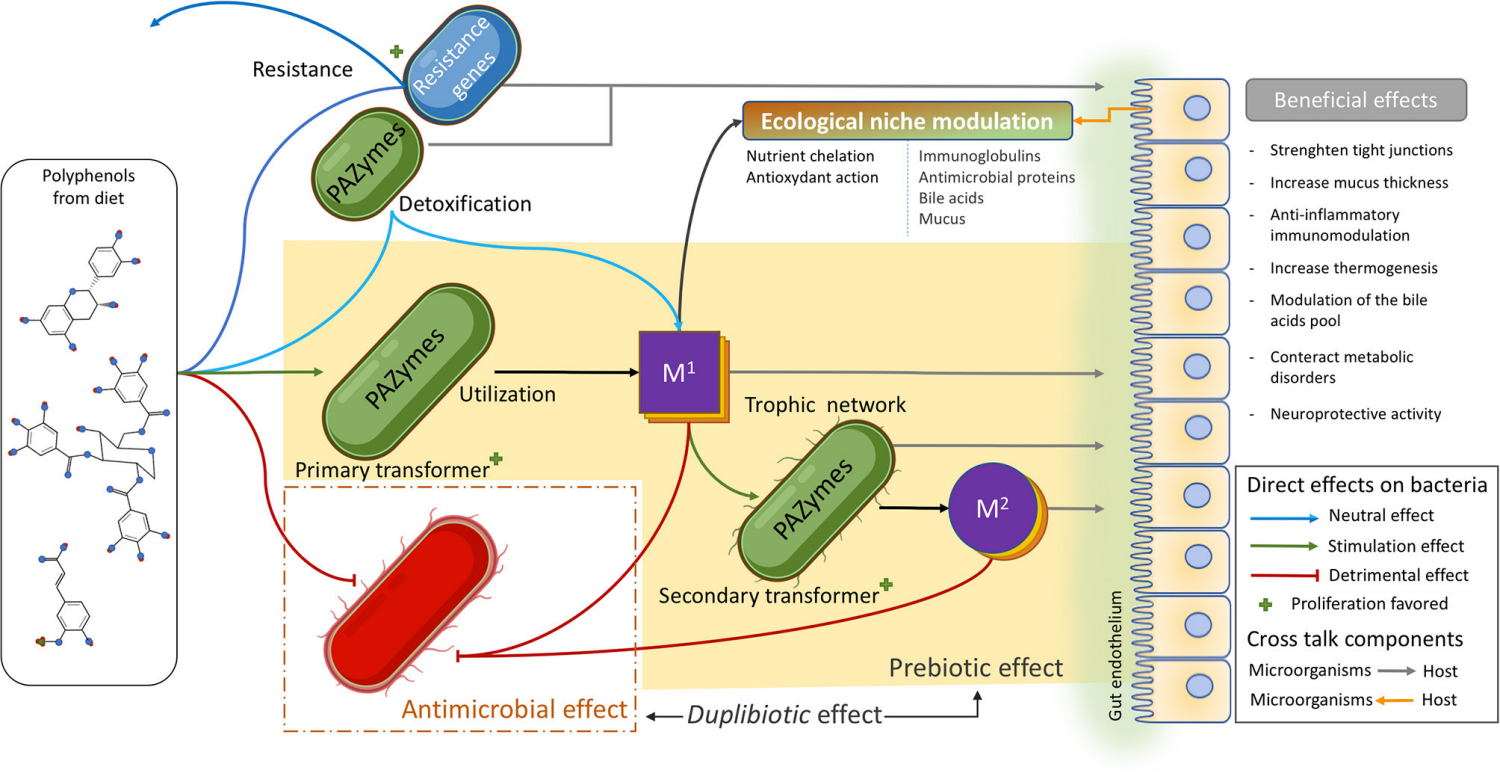
Polyphenol's mode of actions shaping the gut microbiota. (Poly)phenols reaching the gut can exert an important antimicrobial pressure (red lines) on several members of the microbiota. They can also be transformed by a vast and diverse array of microbial (poly)phenol-associated enzymes (PAZymes). Bacteria possessing the required PAZymes can improve their fitness by the metabolic utilization of a given polyphenol and thrive within the (poly)phenol-enriched gut environment (green arrow). This leads to the production of primary bioactive metabolites (M^1^) that can be involved in trophic interactions and further be metabolized by other microorganisms possessing the appropriated PAZymes, releasing secondary metabolites (M^2^), and so on. PAZymes can degrade (poly)phenols without conferring any direct advantage to the bacteria (light blue arrow) in a detoxifying-like effect. The residual product can become a primary bioactive metabolite (M^1^) and enter the trophic network. Other bacteria may not possess PAZymes required for the degradation of a specific (poly)phenolic compound but can instead resist its antimicrobial effect (dark blue arrow). This confers those strains an advantage to take over sensitive competitors. First and second transformers and resistant bacteria, as well as (poly)phenol metabolites (M^1^ and M^2^), can modulate the intestinal ecological niche and induce local and systemic beneficial effects by interacting with the host (gray arrows). In turn, those metabolic changes can also modulate the intestinal ecological niche (orange arrow) in a crosstalk fashion. (Poly)phenol metabolites (M^1^ and M^2^) can also induce an antimicrobial effect (red lines). The yellowish area highlights (poly)phenol prebiotic path process, while the red dashed square identifies the antimicrobial effect of (poly)phenol, both defining the *duplibiotic* effect.

It is suggested that PAZymes repertoire improve the fitness of certain beneficial species, contributing not only to detoxify the bacteria's environment but also enabling these microorganisms to access carbon sources (i.e., β-glucosides) or favoring them metabolically to produce more ATP (i.e., gallate decarboxylase or phenolic acid reductase). These gut bacteria (poly)phenol transformations release a variety of microbial metabolites that, contrary to large polyphenol polymers, easily cross cell membranes (through passive permeation or active transports), reach target tissues, and exert local anti-inflammatory activities. These (poly)phenol-derived metabolites are beneficial as they potentially modulate the immune system while hindering the growth of pro-inflammatory gut bacteria (Figure 5). Certainly, these metabolite-induced outcomes, as demonstrated for their parent compounds, impact the gut microbiota by generating ecological shifts. (Poly)phenol-induced changes in the microbiome (microbial functions, composition, and intestinal environment) (215, 217, 244) imply not only the *duplibiotic* nature of these molecules, but also other potential mechanisms of actions (i.e., as anti-inflammatory and antioxidant agents).

## Concluding Remarks and Future Perspectives

Studies on microbiota-(poly)phenols interaction render insights into targeted mechanisms that can be modified with dietary approaches to counteract gut dysbiosis-associated diseases. Advances in *in-vitro* experimental colonic fermentation have helped to unveil the physiological roles, substrate preferences, and metabolites exchanges of key taxonomic members of the gut microbiota induced by (poly)phenols. In fact, analyses of bioactive phenolic metabolites generated from the action of bacterial PAZymes can predict and allow deciphering microbes-host crosstalk, underpinning the *duplibiotic* nature of dietary (poly)phenols in humans and animals.

In the future, the concept of *duplibiotic* could be extended to other compounds. Plants produce over 100,000 secondary metabolites intended—among other functions—to interact with their own microbiota (272). Thus, it would not be surprising to find in humans' omnivorous diet other phytochemicals which could exert antimicrobial effect or be metabolized by the human gut microbiota. These interactions between the plant molecules and the human gut microbiota can impact both, the microbiota composition, and its activity, as well as the human health itself. Already, several plant alkaloids—berberin (273, 274) and betalain (273, 274)—have been shown to exert antimicrobial and prebiotic-like actions and could be considered *duplibiotics*.

## Author Contributions

MR-D, EP-M, and JL-M have been responsible for the conception of this article and drafting the manuscript. JL-M and MR-D created the figures. EP-M and JL-M constructed the table. DR, YD, and DG mentored and critically reviewed this work. All authors read and approved the final manuscript.

## Conflict of Interest

MR-D is involved in a project funded by DianaFood. EP-M and JL-M research works are funded by a Collaborative Research and Development Grant (RDC), partly funded by Diana-Food and the Natural Sciences and Engineering Research Council of Canada (NSERC). DG is Scientific and Innovation Director at Diana Nova, part of Symrise Nutrition. YD holds an NSERC-DianaFood Industrial Chair on prebiotic effects of fruit and vegetable polyphenols. DR is a principal investigator within the RDC.
